# Genome sequencing as a platform for pharmacogenetic genotyping: a pediatric cohort study

**DOI:** 10.1038/s41525-017-0021-8

**Published:** 2017-05-26

**Authors:** Iris Cohn, Tara A. Paton, Christian R. Marshall, Raveen Basran, Dimitri J. Stavropoulos, Peter N. Ray, Nasim Monfared, Robin Z. Hayeems, M. Stephen Meyn, Sarah Bowdin, Stephen W. Scherer, Ronald D. Cohn, Shinya Ito

**Affiliations:** 1 Division of Clinical Pharmacology and Toxicology, Department of Paediatrics, The Hospital for Sick Children, University of Toronto, Toronto, ON Canada M5G 1X8; 2 Program in Translational Medicine, The Hospital for Sick Children, Toronto, ON Canada M5G 0A4; 3 The Centre for Applied Genomics, The Hospital for Sick Children, Toronto, ON Canada M5G 0A4; 4 Centre for Genetic Medicine, The Hospital for Sick Children, Toronto, ON Canada M5G 1X8; 5 Department of Paediatric Laboratory Medicine, The Hospital for Sick Children, Toronto, ON Canada M5G 1X8; 6 Department of Paediatrics, The Hospital for Sick Children, University of Toronto, Toronto, ON Canada M5G 1X8; 7 Division of Clinical and Metabolic Genetics, The Hospital for Sick Children, Toronto, ON Canada M5G 1X8; 8 Department of Molecular Genetics, University of Toronto, Toronto, ON Canada M5S 1A8; 9 Genetics and Genome Biology Program, The Hospital for Sick Children, University of Toronto, Toronto, ON Canada M5G 1X8

## Abstract

Whole-genome sequencing and whole-exome sequencing have proven valuable for diagnosing inherited diseases, particularly in children. However, usage of sequencing data as a pharmacogenetic screening tool to ensure medication safety and effectiveness remains to be explored. Sixty-seven variants in 19 genes with known effects on drug response were compared between genome sequencing and targeted genotyping data for coverage and concordance in 98 pediatric patients. We used targeted genotyping data as a benchmark to assess accuracy of variant calling, and to identify copy number variations of the *CYP2D6* gene. We then predicted clinical impact of these variants on drug therapy. We find genotype concordance across those panels to be > 97%. Concordance of *CYP2D6* predicted phenotype between estimates of whole-genome sequencing and targeted genotyping panel were 90%; a result from a lower coverage depth or variant calling difficulties in our whole-genome sequencing data when copy number variation and/or the CYP2D6*4 haplotype were present. Importantly, 95 children had at least one clinically actionable pharmacogenetic variant. Diagnostic genomic sequencing data can be used for pre-emptive pharmacogenetic screening. However, concordance between genome-wide sequencing and target genotyping needs to be characterized for each of the pharmacologically important genes.

## Pediatric medicine: Diagnostic sequencing offers useful pharmacogenetic data, too

Genome sequencing, in addition to helping diagnose disease, can inform medication decisions and improve drug safety for children. Ronald Cohn, Shinya Ito and colleagues at the Hospital for Sick Children in Toronto, Canada, studied a cohort of 98 pediatric patients who had undergone whole genome sequencing to help diagnose their unexplained congenital malformations or neurodevelopmental disorders. The researchers looked for 67 DNA variants found in 19 genes with known effects on drug responses. They used targeted genotyping to assess the accuracy of the sequence data. Sequencing proved to be more than 99% accurate for all but one of the pharmacologically important genes, showing the power of diagnostic genomic sequencing to identify DNA variants in children that affect medication safety and effectiveness. However, the accuracy of the method may need to be validated for each relevant gene.

## Introduction

Over the last decade, there has been significant growth in the use of genetic information to individualize clinical care. Pharmacogenetic testing in particular has seen a surge in interest because of increased patient safety awareness programs and the opportunity to identify patients who are likely to respond to certain medications and/or those in whom there is a high probability of developing severe adverse drug reactions attributed to individual genetic variants.^1, 2^ However, the majority of these studies are in adult cohorts. Although pharmacogenetic pediatric research has already yielded promising results,^3^ advances in genome-sequencing technologies now provide the opportunity to broaden and deepen the scope of pediatric pharmacogenetics as a pre-emptive medication safety screening tool.

Currently, most laboratories conducting pharmacogenetic testing use targeted genotyping technologies to clinically screen for specific variants with well-characterized drug-gene interactions. Examples of these technologies include single or multiplexed PCR assays using Taqman hydrolysis probe chemistry (Life Technologies), mass spectrometry (Agena Biosciences), bead-based immunoassay testing (Luminex), and microarrays (Affymetrix).^4, 5^

In addition to providing a powerful tool for diagnosing inherited disorders in childhood,^6^ whole-exome sequencing (WES) and whole-genome sequencing (WGS) carry the promise to identify clinically relevant pharmacogenetic variants. Mining sequence data for pharmacogenetic variants is particularly appealing in pediatric patients as it serves as an example of predictive and individualized medicine. Yet, in order to use this data confidently, it needs to be established whether variants in pharmacogenes are adequately covered and accurately genotyped from these genome-sequencing platforms. Previous comparisons between exome/genome and targeted genotyping show potential in this area,^7, 8^ however, performance assessments of those platforms and estimating copy number variation (CNV) in pharmacogenes in the same pediatric patient cohort have not been explored.

Drawing from a cohort of 98 children who underwent WGS for diagnostic purposes,^6^ we examined the coverage of WGS along with concordance between WGS and targeted genotyping for a set of 67 single-nucleotide polymorphism (SNP) and indel variants in 19 pharmacogenes. We also compared estimates of *CYP2D6* gene copy number between WGS and targeted genotyping. Although not the primary objective, we investigated the utility of WES for pharmacogenetic analysis for 12 samples in the cohort because these data were available. SNP selection was based on published drug—gene dosing guidelines as well as known drug—gene interactions with potential for future pharmacogenetic guidelines. Furthermore, we explored whether variants examined carried the potential to inform future medication decisions and thus provide an opportunity to enhance patient safety.

## Results

### Extraction of pharmacogenetic data from various testing platforms

To determine the accuracy of pharmacogenetic data extracted from one genomic-sequencing platform (Complete Genomics), we compared the genotype calls for 67 pharmacogenetic loci (Table 1) for 98 subjects to genotypes generated using two targeted genotyping panels. We used the targeted genotyping data as a benchmark to assess variant calling and to predict copy number status of the *CYP2D6* gene. Following this, we predicted phenotype status for metabolizer genes. We genotyped 98 subjects with the iPLEX® ADME *CYP2D6* Panel, analyzing 29 *CYP2D6* SNP and indel variants, with a 99.8% success rate (2835 genotypes). For the remaining 38 variants (in 18 other genes), we designed a custom iPlex panel in a 2-well assay (Table 1). The 98 subjects were successfully genotyped for all 38 variants in this set, except for one position (*ABCG2*, rs2231137) for one sample.

**Table 1:** Overview of the 67 variants examined and compared

Reference SNP (haplotype)	Gene
rs1801131; rs1801133	*MTHFR*
rs67376798; rs3918290	*DPYD*
rs12248560, rs28399504, rs41291556, rs17884712, rs4986893, rs4244285	*CYP2C19*
rs1799853, rs9332131, rs1057910, rs28371686	*CYP2C9*
rs1800497	*ANKK1*
rs1954787	*GRIK4*
rs2306283; rs4149056	*SLCO1B1*
rs9923231	*VKORC1*
rs2108622	*CYP4F2*
rs12979860	*IFNL3*
rs1051266	*SLC19A1*
rs4633; rs4818; rs4680	*COMT*
rs1135840, G4125_4133 T (rs765776661), rs28371731 (rs4987144), rs72549346, rs72549347 (rs147960066), rs2837172, rs72549349, rs5030867, rs16947, rs5030656 rs72549351, rs72549352, rs35742686, rs72549353 (rs758320086), rs72549354, rs72549356 (rs553846709), rs3892097, rs5030865, rs5030655, rs1058164, rs61736512, rs28371706, rs5030863 (rs201377835), rs72549357 (rs774671100), rs5030862, rs1065852, rs769258, rs28735595, rs1080985	*CYP2D6*
rs2228001	*TMEM*
rs2231142, rs2231137	*ABCG2*
rs1142345, rs1800584, rs1800460, rs1800462	*TPMT*
rs1061235 (HLA- A* 31:01), rs2395029 (HLA-B* 57:01)	*HLA*
rs1045642, rs2032582, rs1128503	*ABCB1*
rs776746	*CYP3A5*

**Fig. 1:**
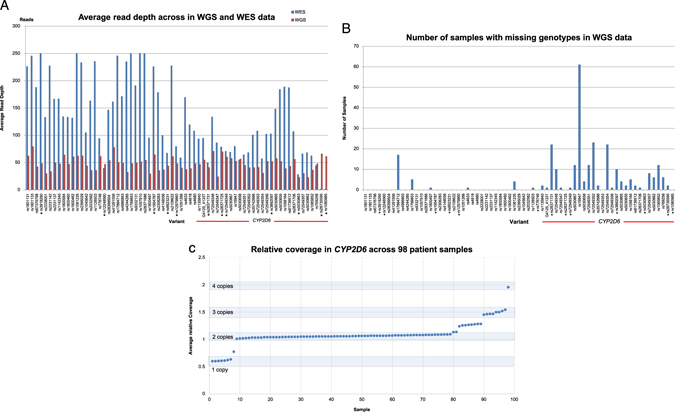
**a** Average read depth across the 98 study samples in WGS (complete genomics) and WES (Illumina HiSeq) data. Project loci are displayed by Reference SNP and in order of genomic coordinate (within the gene), although not to scale. Variants denoted with an *asterisk* (*) are located in introns. **b** Number of samples with missing genotypes in WGS data found across the 98 study samples for each genomic coordinate. Variants denoted with an *asterisk* (*) are located in introns. **c** Relative coverage for a 6 kb window encompassing *CYP2D6* gene across 98 patient samples. An average relative coverage of 1 in *CYP2D6* is assigned a copy number of 2. The *shared boxes* (and number above them) denote the assigned *CYP2D6* copy number

Analysis of the WGS data revealed an average depth of coverage of 20X or greater for all 67 loci (average range 21.6X—79.5X: Fig. 1a) across the 98 subjects. Passing quality calls were observed for 96% of the data (6312 of the 6566 positions; Fig. 1b). This metric was over 99% in non-*CYP2D6* positions. Another genomic position (CYP2C19*9, rs17884712) with low- quality calling was observed in a portion of the study samples (17/98).

### CYP2D6 data analysis

Seven positions in *CYP2D6* were missing calls in more than 10 patients in WGS due to low-confidence calling (Fig. 1b); rs16947, a common *CYP2D6* variant found in the CYP2D6*2 haplotype among others, was the most frequently missed position (60 of the 98 subjects). However, in subjects called successfully at this position, rs16947 had an average depth of coverage of approximately 50. For subjects missing a call for rs16947 and/or additional variants, the most likely *CYP2D6* diplotype was manually and individually assigned by using the *CYP2D6* star nomenclature^9^ (Table S1). However, a diplotype could not be assigned to 14 of the 98 subjects due to missing calls in key positions. Six of these 14 subjects (Patient ID: 1022, 1031, 1066, 1075, 1092, 1108) carried rs3892097 variant defining the CYP2D6*4 haplotype and seven subjects (Patient ID: 1039, 1053, 1063, 1086, 1090, 1093, and 1096) had *CYP2D6* copy number gains or losses (described below). One sample (Patient ID 1093) carried a combination of both (copy number gain + CYP2D6*4).

Lastly, we looked at coding variants in the *CYP2D6* gene outside of the set examined here (Table S3). We observed three synonymous and ten non-synonymous variants that were individually examined for potential effect on the activity of *CYP2D6*. Twelve of the 13 variants were observed in single individuals. The majority of variants, although some predicted to be deleterious, did not change the metabolizer status of *CYP2D6* for the subject. However, two variants (P41L and R329L) were investigated further because of their potential to affect metabolizer status depending on the allele they occur in. Inspection of the BAM files at these coordinates suggested that the P41L variant is on the *4 allele (P34S), although this is supported by a very small number of reads that span both variants. The phase of the R329L variant could not be determined from the BAM file. These variants remain of unknown clinical significance.

### Concordance of genotype calls and copy number estimation in the WGS data as compared to targeted genotyping

Between the WGS and targeted genotyping data sets, there were six discordant genotypes in three genes (one in *CYP2C9*, one in *HLA-A* and four in *IFNL3*) (Table S2). However, the 254 missing or poor quality calls (the majority in *CYP2D6*) limited a complete comparison of these platforms.

CNV in *CYP2D6* is relatively common, and has been established as important accompanying information in *CYP2D6* typing.^10^ When we examined *CYP2D6*-overlapping CNVs in the Complete Genomics cnvSegmentsDiploidBeta and high ConfidenceSVEventsBeta files, only one sample was flagged as having a copy number gain of *CYP2D6*. We instead extracted relative coverage (defined as normalized coverage level under a diploid model, a value of “1” being 2-copy) for a 6 kb window containing *CYP2D6*. We observed an average relative coverage deviation of more than 0.2 from the value of 1 in 25 samples (Fig. 1c), suggesting a possibility of CNV. Seven samples displayed an average relative coverage deviation of close to 0.5 (heterozygous deletion or copy number of 1), while eight samples had values of close to 1.5 (duplication or copy number of 3). One individual had an average relative coverage deviation of 1.96 (copy number of 4). A subset of nine samples was inconclusive for copy number by this method, as they exhibited intermediate values of average relative coverage (between 0.5 and 1 or between 1 and 1.5) (Fig. 1c). Interestingly, the majority of these samples (7/9) contain the CYP2D6*4 variant rs3892097 (Table S1). In heterozygous three-copy samples, we manually inspected the reference and alternative read counts at informative positions to identify the duplicated allele. We were able to determine the duplicated allele for three samples (Patient ID: 1012, 1018, 1029) in this way. The read count at these informative positions was 75 or greater in all three samples (Table S1).

The Agena Typer software identified seven samples with one copy of *CYP2D6*, 81 samples with two copies, nine samples with three copies and one sample with four copies of *CYP2D6*. However, in heterozygous three-copy samples we could not confidently deduce the duplicated allele by manual inspection of allele-specific peak heights of informative SNPs.

There were two discordant samples in the estimation of the *CYP2D6* CNVs between WGS and targeted genotyping data: (1) one sample (Patient ID: 1088) was estimated to be one copy with WGS, but two copy with targeted genotyping panel and (2) one sample (Patient ID: 1075) was estimated to be two copy with WGS but three copy with the targeted genotyping panel. Of the nine samples (Patient ID: 1009, 1025, 1031, 1043, 1068, 1070, 1072, 1074, 1112) inconclusive for genomic copy number of *CYP2D6* in WGS data, one sample (Patient ID: 1074) was estimated to be one copy while the other eight samples were classified as a two copy using the targeted genotyping panel (Table S1).

### Concordance of genotype calls in WES as compared to WGS and targeted genotyping platforms

Data from WES were available for 12 samples from the same patient cohort and so was included in our analysis. In the 12 WES samples, the 67 loci were sequenced to an average depth of 140X, and there were no missing calls for variants in exonic regions (Fig. 1a). Two positions over 1 kb upstream of exon 1 (*CYP2D6*: rs28735595, rs1080985) were not covered by this WES data set, however, all other *CYP2D6* positions were successfully genotyped. Considering positions with passing quality calls (*n* = 6312), the concordance between WGS, WES, and the targeted genotyping panel was high (> 99.9%).

### Clinical utility of pharmacogenetic data using genome-wide-sequencing platforms

**Fig. 2:**
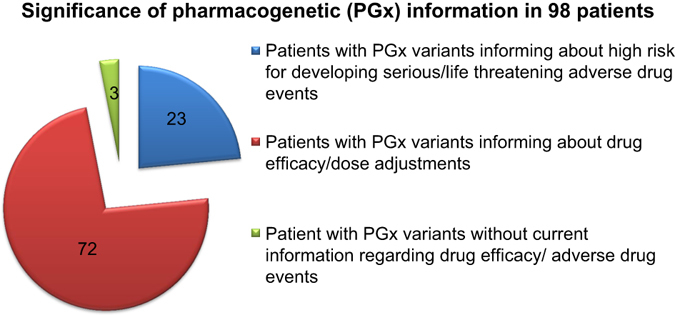
Significance of pharmacogenetic (PGx) information in 98 subjects. Based on published guidelines, mined PGx data from targeted genotyping and WGS platforms were subdivided into three different categories by considering the significance of extracted information on drug metabolism and drug response

In order to gain insight into the potential clinical utility of pharmacogenetic data extracted from genome-wide-sequencing platforms, we merged genetic data from WGS and targeted genotyping platforms for all 98 children and used published pharmacogenetic guidelines in order to review drug-gene interactions (Table 2). In the combined data, we were able to predict phenotypes of genes involved in metabolism and elimination of medications for all 98 subjects (Fig. S1). We detected at least one clinically relevant variant in 95 of the 98 subjects that could point to an individualized drug selection and/or dosing adjustment (Fig. 2).

**Table 2:** Overview of interrogated drug-gene pairs

Drug	Indication	Benefits to testing	Gene	Guidelines
Aripiprazole	Psychiatry	Improves drug efficacy and safety	CYP2D6	CPIC
Atomoxetine	Neuropathic pain*			DPWG
Desipramine*				
Duloxetine*				
Fluvoxamine				
Haloperidol Nortriptyline				
Paroxetine				
Venlafaxine				
Citalopram, Esctialopram Sertraline	Psychiatry	Improves drug efficacy and safety	CYP2C19	CPIC
Amitriptyline*	Psychiatry	Improves drug efficacy and safety	CYP2D6	CPIC
Clomipramine	Neuropathic pain*		CYP2C19	
Doxepin				
Imipramine*				
Trimipramine				
Codeine	Pain	Improves drug efficacy and safety	CYP2D6	CPIC
Oxycodone		Prevents serious adverse drug reactions		
Tramadol				
Clopidogrel	Cardiology	Improves drug efficacy and safety	CYP2C19	CPIC
	Neurology (anticoagulant)	Prevents futile use of the drug in genetic non-responders		
Warfarin	Cardiology	Improve drug efficacy and safety	CYP2C9	CPIC
	Neurology (anticoagulant)	Prevents serious bleeding events or stroke while achieving therapeutic effects faster in initial dosing	VKORC1	
Flecainide	Cardiology	Improves drug efficacy and safety	CYP2D6	DPWG
Propafenone	(Antiarrythmic)			
Simvastatin	Cardiology Internal medicine (antihyperlipidemic)	Improves drug safety Prevents drug-induced myopathy	SLCO1B1	CPIC
Carbamazepine	Neurology	Improves drug safety	HLA-A*3101	CPIC
	Psychiatry	Prevents serious and sometimes life-threatening hypersensitivity reactions	CYP2C9	
	Neuropathic pain			
Abacavir	Infectious diseases (HIV, AIDS)	Improves drug safetyPrevents serious and sometimes fatal hypersensitivity reactions	HLA-B*5701	CPIC
Boceprevir,	Infectious diseases	Improves drug efficacy	IFNL3	CPIC
Peginterferon α 2a/2b	(Hepatitis C)	Prevents futile use of the drug in genetic non-responders		
Ribavirin				
Telaprevir				
Esomeprazole	Gastroenterology	Improves drug efficacy	CYP2C19	DPWG
Lansoprazole				
Omeprazole				
Pantoprazole				
Azathioprine	IBD, cancers,	Improves drug safety	TPMT	CPIC
6-Mercaptopurine	Autoimmune disorders	Prevents serious and sometimes life-threatening myelotoxicity		
Thioguanine				
Tamoxifen	Cancer	Improves drug efficacy	CYP2D6	DPWG
		Prevents futile use of the drug in genetic non-responders		
Capecitabine	Cancer	Improves drug efficacy and safety	DPYD	CPIC
Fluorouracil		Prevents serious and sometimes life-threatening reactions		
Tegafur				
Tacrolimus	Graft- vs.- Host disease, Autoimmune disorders	Improve drug efficacy	CYP3A5	CPIC

* links medication to alternative indication

We considered the relevance of the pharmacogenetic information for each individual and divided the pharmacogenetic dosing guidelines into medical subspecialties. Our analyses showed that 70% of patients from this cohort would specifically benefit from dose adjustments in drugs that are mainly used in cardiology and psychiatry, followed by infectious diseases (54%), neurology (42%), gastroenterology (30%), transplant (25%), pain (9%), and oncology (8%). Assessment of the drug–gene interactions revealed that 23% of our patient samples had an increased risk of developing serious adverse events in drugs used in neurology (9%), oncology (8%), infectious disease (6%), and pain management (3%) (Fig. S2).

Our findings highlight the potential for proactive pharmacogenetics using genome-sequencing data to prevent exposing individuals to an increased risk of developing adverse drug events or therapeutic failure of medications with known drug–gene interactions over a lifetime.

## Discussion

Genomic-sequencing technologies are now being translated into clinical care and have significantly improved the ability to establish diagnoses in inherited disorders. Since most of these disorders manifest in childhood, the role of genomic-sequencing technologies in pediatrics is particularly poignant. Genome sequencing carries a significant promise for the field of pharmacogenetics, an area that provides the basis to prevent severe side effects and ineffective drug treatments.^11^

It has previously been reported that genomic-sequencing data can be mined for pharmacogenetics variants.^12, 13^ Several studies of genomic-sequencing platform comparisons involving some pharmacogenetic variants have been reported.^7, 8^ These studies generally conclude that concordance between sequencing platforms is high for common genetic variants in coding regions. In our study, we systematically analyzed 67 SNP and indel variants with reported clinical pharmacogenetic relevance across three different platforms (WGS, WES, and targeted genotyping) in the same patient cohort. Our goal was to not only confirm that pharmacogenetic variants can be called from genomic-sequencing data sets, but also to determine (1) how well each variant was covered across samples; (2) the quality of the variant calls, and (3) the accuracy of these variants as compared to one standard method of targeted genotyping.

We observed a high concordance (> 99%) of SNP and indel variants called from WGS with those obtained from our targeted genotyping panel. However, we also found WGS was not able to accurately genotype several positions in *CYP2D6*. In particular, genotype information for the rs16947 SNP that defines CYP2D6*2-related haplotypes was absent in the majority of subjects (60/98) with WGS data. This required manual derivation of diplotypes based on the remaining calls and could be done with confidence in many cases. In a clinical setting, these individuals would require reflexed testing using targeted genotyping of the *CYP2D6* gene. We found that individuals with an rs3892097 variant (CYP2D6*4) were more likely to have ambiguous calling (no call or only one allele confidently called) for several *CYP2D6* positions. Structural variants involving the *4 haplotype are well known^14, 15^ and PCR-based testing for the various configurations and hybrids would be a logical follow-up in future. Our findings are also consistent with in silico modeling showing that short reads of *CYP2D6* multi-align to the highly similar *CYP2D7* and *CYP2D8* genes,^16^ leading to the reasonable assumption that WGS variant calling in *CYP2D6* will be platform-dependent because of variations in sequence coverage due to chemistry, read length and downstream bioinformatics tools.^17^ At this time, we recommend pursuing additional targeted testing for the *CYP2D6* gene to confirm WGS data. Overall, six discordant genotype calls (both false positive and false negative) were observed between WGS (Complete Genomics) and our targeted genotyping panel in three different genes (*CYP2C9*, *HLA-A*, and *IFNL3*), which are currently being investigated (Table S2).

In our study, we identified duplications or deletions in the *CYP2D6* gene in 17 out of 98 subjects, a prevalence which mirrors known CNVs of *CYP2D6* in the US population.^18^ In three cases of copy number gain (Patient ID: 1012, 1018, 1029), we were able to manually inspect the ratios of reference and alternate allele reads at informative positions in the whole-genome data to deduce the duplicated allele. This may indicate an advantage of sequencing over genotyping, as the same could not be done confidently from the genotyping data (using peak height ratios). In five samples with a copy number gain (Patient ID: 1012, 1018, 1020, 1061, 1085) the prediction of *CYP2D6* metabolizer status changed when compared to an individual of the same genotype without a duplication (Table S1). Although *CYP2D6* copy number could be determined in most cases from the WGS data, copy number status was ambiguous in nine of the 98 samples examined. We speculate that mapping issues, as discussed above, complicate copy number determination. Alternatively, these individuals may harbor structural variations of *CYP2D6* such as *CYP2D6/CYP2D7* hybrids or complex tandem arrangements.^14–16^

Recent improvements in cost and accuracy of WES have made it feasible to use it as a molecular diagnostic tool for patients referred to evaluation of suspected genetic conditions.^19^ We, therefore, examined WES data (already available) from 12 individuals of our patient cohort and compared the variant calling data for the 67 positions to targeted genotyping data. Although we anticipated similar variant calling issues as observed in the WGS data, interestingly, WES variant calling in *CYP2D6* was highly concordant to targeted genotyping and no position exhibited similar rates of missing data. Two SNPs outside of exons (rs28735595, rs1080985) that are traditionally examined to assign *CYP2D6* haplotypes were not captured (Fig. 1a). We do note, however, that the upstream variant rs12248560 (CYP2C19*17) was covered at a sufficient depth for variant calling (average 47X) indicating that some commercial bait sets may be supplemented to capture intronic regions.^20^ Current algorithms for CNV detection from WES data are limited in their performance,^21, 22^ therefore, we did not attempt to detect CNV’s from WES data.

During the last centuries, medical practice has undergone a significant transformation. In the 19th century, the focus was mainly to treat symptoms, followed by treating diseases in the 20th century. Now, as we are at the beginning of the 21st century, focus is shifting toward predictive and pre-emptive treatments of symptoms and diseases. This allows for a shift from a late curative paradigm to an early pre-emptive one, which is becoming increasingly possible.^23^ Pharmacogenetics will play a critical role in this paradigm shift toward predictive and pre-emptive medicine and in order to maximize its benefit it will need to be employed in the pediatric population. Although, when a genotype–phenotype relationship is identified, the effect of developmental factors such as change of enzyme activity has to be considered, as it greatly affects drug response and tolerance in children.^24^ Nevertheless, genetic determinants of drug response remain stable throughout life and thus offer great promise to individualized drug therapy.^25^ Here we demonstrated that out of 98 samples, 95 samples harbored pharmacogenetics variants with actionable clinical results as established by pharmacotherapeutic evidence-based, peer-reviewed published guidelines from the Clinical Pharmacogenetics Implementation Consortium (CPIC), the Dutch Pharmcogenetic Working Group (DPWG) and the Food and Drug Administration (FDA).^26^ Our data are consistent with the results described previously.^25^ Furthermore, 23 samples of these individuals carried pharmacogenetic variants, which are known to have a high probability of developing serious/life threatening adverse drug events (Fig. 2; Fig. S2). These findings lend weight to the view that although there is a relatively small set of medications for which pharmacogenetics offers actionable data, future prescribing for these individuals could be optimized if genetic testing were more widely and appropriately deployed in the clinic.^27^ Currently, only a few pediatric pharmacogenomic test kits are commercially available and used in clinical practice.^28^

**Fig. 3:**
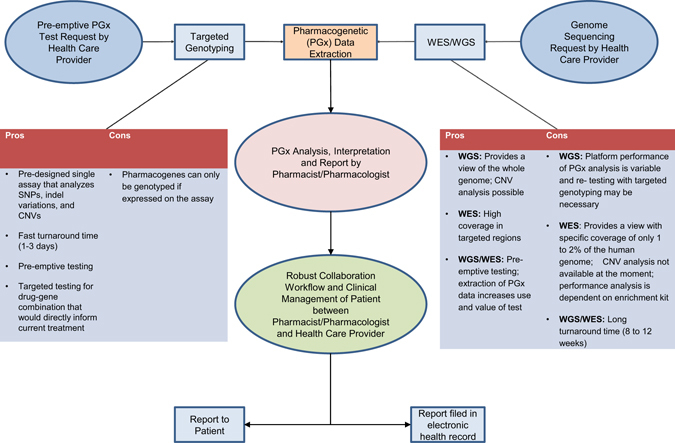
Workflow of incorporating PGx data into clinical care. This figure presents two clinical approaches how pre-emptive interpretation of pharmacogenetic variants can be incorporated into the medication prescribing process in the future. Pharmacogenetic data can be extracted by either a comprehensive pharmacogenetic genotyping testing panel made available to the health-care public or by genome sequencing currently used in clinical care of pediatric and adult patients. In both scenarios pharmacogenetic trained clinical pharmacists and/ or pharmacologists should be involved in assisting to interpret the results and communicate back to the ordering health-care provider and/or patient through a robust and collaborative partnership

As genomic-sequencing technologies continue to improve regarding read length, data analysis and variant interpretation, pharmacogenetic testing should be considered in various primary care, outpatient and inpatient settings. We propose a two-pronged approach to the collection of pharmacogenetic data in the clinic and application to the medication prescribing process. In one arm, a conventional genotyping testing panel, for pharmacogenes with published dosing guidelines, should be made available to primary care and physician office visits, as well as hospital inpatient, outpatient, and emergency room visits as part of a laboratory blood work. In a clinical setting, where results are often required within days of administering diagnostic tests, targeted genotyping is advantageous for its cost efficiency, easy data analysis, and fast turn-around time as it directly informs applicable medication treatment choices.^29^ Currently, the extraction of pharmacogenes from genomic sequencing is reserved for pre-emptive information seeking individuals who are undergoing a genomic diagnostic test for an indication unrelated to pharmacogenetics. Our study provides evidence that genomic-sequencing data can also be used to extract pharmacogenetic variants. However, it is important to note that variant calling, especially in the *CYP2D6* gene, could be challenging depending on sequencing platform used. For *CYP2D6*, manual interpretation of WGS data in the form of targeted CNV analysis, inspection of allelic read depth and decision-making surrounding missing markers, was necessary here with data generated on the Complete Genomics platform. Further PCR-based testing could also be done on some subjects to determine if they harbor structural variants of *CYP2D6* not detectable by the methods here. As other WGS technologies become accessible, we recommend rigorous validation of each platform for pharmacogenetic variant calling. In whole-exome data, while most pharmacogenetics markers of interest had adequate coverage for variant calling, algorithms for copy number determination from these data are not fully developed. In the case of *CYP2D6*, the copy number status is an integral part of typing an individual so this testing would need to be done separately via quantitative PCR or other method. In all cases, pharmacogenetics-trained clinical pharmacists and/or pharmacologists should be involved in result interpretation and provide a report that highlights medically actionable and clinically relevant data to the primary physician, thus allowing the health-care provider to make effective and safe treatment decisions for adults as well as for pediatrics. Furthermore, it will be important to ensure that this information remain part of any electronic medical health record, improving outcomes for drug-mediated treatments over a lifetime (Fig. 3).

## Materials and Methods

### Study cohort

Study participant and recruitment details are described elsewhere.^6^ Briefly, 98 children were recruited prospectively from the Genome Clinic in the Division of Clinical and Metabolic Genetics at Toronto’s Hospital for Sick Children over a 9-month period (September 2013–May 2014). DNA of the study participants was extracted from peripheral blood using the QIAsymphony DSP DNA Midi Kit on the QIAsymphony SP instrument. This study was approved by the Research Ethics Board at The Hospital for Sick Children and informed consent was obtained from all participants. Methods were performed in accordance with relevant regulations and guidelines.

### Testing platforms

#### Whole-genome sequencing

Genomic DNA was sent to Complete Genomics (Mountain View, CA) for WGS as described previously.^9^ Raw sequence reads were reassembled against a reference genome (GRCh37) and variant calling was completed (and assigned a designation of ‘‘pass’’) using Complete Genomics assembly pipeline 2.4 (ref. 30). All samples passed internal Complete Genomics sample checks. Sequence results were received on hard drives and consisted of raw data plus variant calls in the form of SNP, indels, structural variants, and copy number variants. Custom VCF files were generated from the Complete Genomics files for reference and variant calls for the 67 genomic coordinates of interest (Table 1). Additional coding variants in the CYP2D6 gene outside of the set were examined from the masterVar files. Variants were filtered for passing quality. Additionally, variants which are assigned to be part of the subject’s haplotype,^9^ but are not genotyped in the panel, were excluded. Variants in the final list were examined for effect, if the variant is associated with a *CYP2D6* allele^9^ and whether the variant would change the metabolizer status of the subject compared to their initial assignment. BAM files were used to confirm the phase of the variant. Variants were not validated with another method and predictions of function are theoretical only. CNV from Complete Genomics are detected through both read depth and paired-end sequencing and provided in cnvSegmentsDiploidBeta and highConfidenceSVEventsBeta files, respectively described in ref 31. We searched the cnvSegmentsDiploidBeta file for segments overlapping *CYP2D6* (GRC37/hg19 chr22:42,522,501–42,526,883). Separately, we extracted relative coverage for a 6 kb region (three 2 kb windows spanning chr22:42,522,000–42,528,000).

#### Whole-exome sequencing

Exome capture and sequencing for 12 samples from the study cohort was performed in the Genome Diagnostics Laboratory at The Hospital for Sick Children. Briefly, exome capture was carried out using the Agilent SureSelectXT Clinical Research Exome target enrichment kit from 500 ng of genomic DNA. Sequencing (2X 100 bp paired end) was carried out on Illumina HiSeq2500 on rapid mode using V1 sequencing chemistry following the manufacturer’s instructions. Base calling was performed using CASAVA v1.8.2 and reads were mapped to the hg19 reference sequence using the BWA-backtrack algorithm from BWA v0.7.8. Duplicate reads were removed using MarkDuplicates from Picard v1.79. Local read realignment around indels, base quality score recalibration, and variant calling with GATK v2.8.1. SNP calls were subjected to variant quality score recalibration. Variant annotation was performed using annovar and custom scripts.

#### Targeted genotyping

All samples were genotyped for 67 positions (Table 1) using iPLEX Pro chemistry on the MassARRAY® Analyzer 4 System (Agena Biosciences, San Diego, CA, USA). Twenty-nine SNP and indel variants as well as copy number status of the *CYP2D6* gene were analyzed using the Agena iPLEX® ADME *CYP2D6* Panel v1.0, which is a 3-well assay combining genotyping for SNP and indel variants as well as five assays to determine genomic copy number. This genotyping platform and assay have been previously shown to accurately genotype pharmacogenetic loci in Coriell reference samples.^32^ *CYP2D6* diplotypes and CNV calling for MassArray data were determined using the Agena PGx Report 2.0 Reporter plugin for the Typer Analyzer software (Agena Bioscience). *CYP2D6* copy number was estimated from the five copy number assays that are integrated in the *CYP2D6* genotyping panel, which is calculated from informative polymorphisms between *CYP2D6* and *CYP2D7*. Variants in the remaining 18 genes in this study were also typed on the MassARRAY® Analyzer 4 System with custom-designed primers using a combination of Agena’s Assay Design Suite (ADS) and Primer3 (ref 33).

#### Comparison among WGS, WES, and MassArray-based targeted genotyping

Each position was manually examined for quality and read depth (in the case of sequencing) and targeted genotyping were also manually inspected. We examined genotype concordance between WGS, WES, and targeted genotyping, and CNV concordance between WGS and targeted genotyping only.

#### Phenotype prediction of samples based on consolidated data

The clinical utility of pharmacogenetics data was examined for all 98 samples, based on published guidelines established by the Clinical Pharmacogenetics Implementation Consortium (CPIC), Dutch Pharmacogenetic Working Group (DPWG) and in US. Food and Drug Administration (FDA) label recommendations. Information regarding the effects of allelic variation on dosing guidelines can be found at the Pharmacogenomics Knowledgebase (PharmGKB) website.^26^

We used the Human Cytochrome P450 Allele Nomenclature Database^9^ to define variant alleles in *CYP2D6*, *CYP2C9*, *CYP2C19*, and *CYP3A5* genes and their effect on their respective *CYP* protein. Furthermore, phenotype assignments such as poor, intermediate, extensive, and ultrarapid metabolizers were determined by utilizing the corresponding published CPIC guidelines^34–40^ and for *CYP2D6*, the activity score system as described elsewhere.^41^ We utilized published guidelines available on the PharmGKB website to determine the influence of polymorphic variations in the remaining genes (HLA-A*31:01, HLA-B*57:01, *IFNL3*, *SLCO1B1*).^42–45^

Also, based on the genotype we subdivided the 98 pediatric sample cohort into three different categories by considering the significance of the variants on predicted drug metabolism and drug response: Category 1 for individuals with currently no pharmacogenetics variants of interest; Category 2 for individuals with variants that would benefit from pharmacogenetics-guided dosing; and finally Category 3 for individuals that carry pharmacogenetic variants, that are associated with developing a serious/life threatening response to a particular drug if treated with such.

### Data availability statement

WGS data are deposited in the European Genome-phenome Archive (www.ebi.ac.uk/ega/) under accession number EGAS00001001623. In addition, the data that support the findings of this study are available from the corresponding author upon reasonable request.

## Electronic supplementary material

Supplementary figure 1

Supplementary figure 2

Supplementary Table 1

Supplementary Table 2

Supplementary Table 3
