# Supplementary material for: Genome sequencing as a platform for pharmacogenetic genotyping: a pediatric cohort study
Source: NPJ Genom Med. 2017 May 26;2:19. doi: 10.1038/s41525-017-0021-8 (PMC5677914; doi:10.1038/s41525-017-0021-8)
Supplement: Supplementary file 1 — Supplementary figure 1 [file 41525_2017_21_MOESM1_ESM.pptx]

## Slide 1
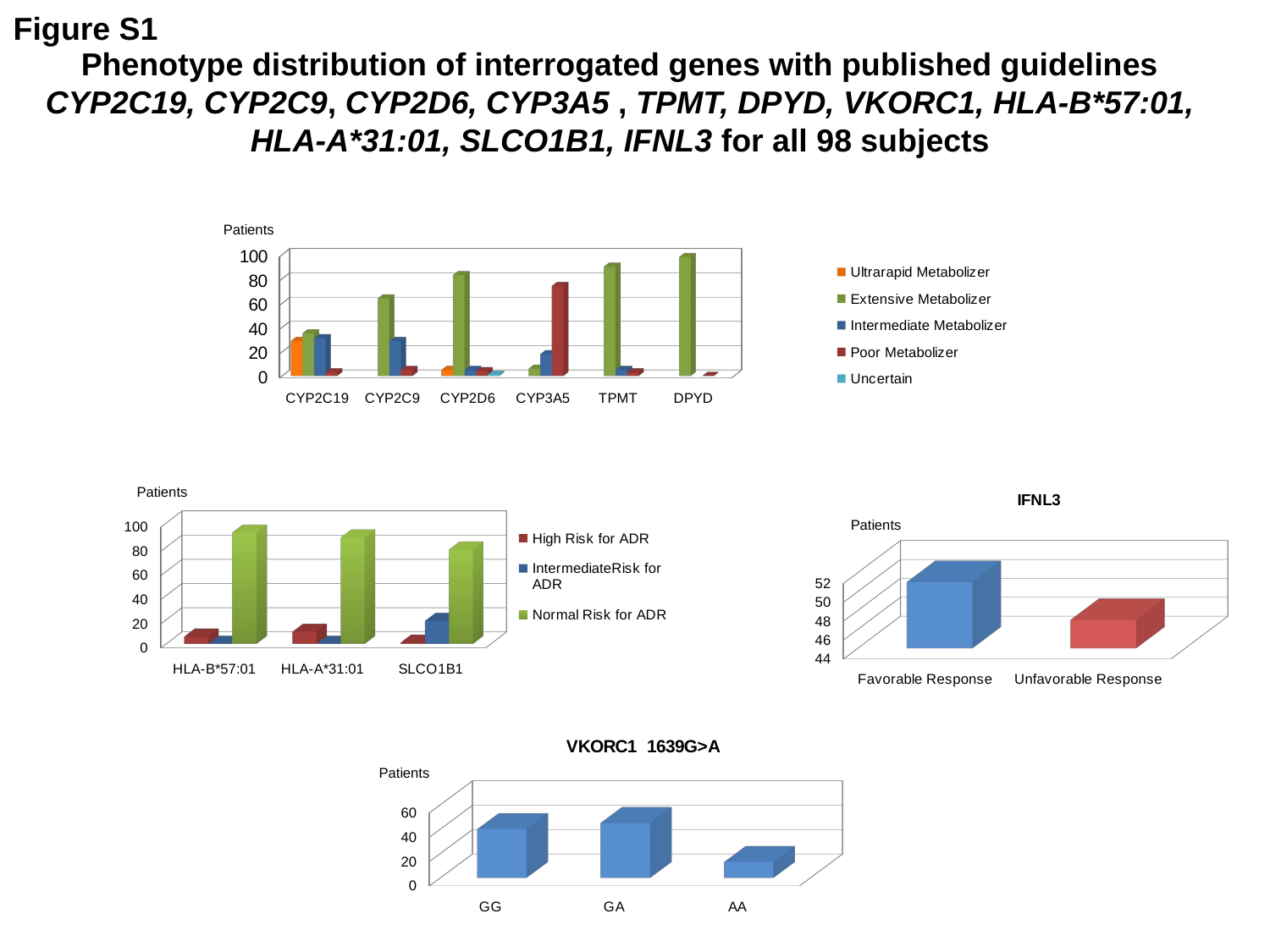

Figure S1
# Phenotype distribution of interrogated genes with published guidelines CYP2C19, CYP2C9, CYP2D6, CYP3A5 , TPMT, DPYD, VKORC1, HLA-B*57:01, HLA-A*31:01, SLCO1B1, IFNL3 for all 98 subjects
[unsupported chart]
[unsupported chart]
[unsupported chart]
[unsupported chart]
