# Supplementary material for: Genome sequencing as a platform for pharmacogenetic genotyping: a pediatric cohort study
Source: NPJ Genom Med. 2017 May 26;2:19. doi: 10.1038/s41525-017-0021-8 (PMC5677914; doi:10.1038/s41525-017-0021-8)
Supplement: Supplementary file 2 — Supplementary figure 2 [file 41525_2017_21_MOESM2_ESM.pptx]

## Slide 1
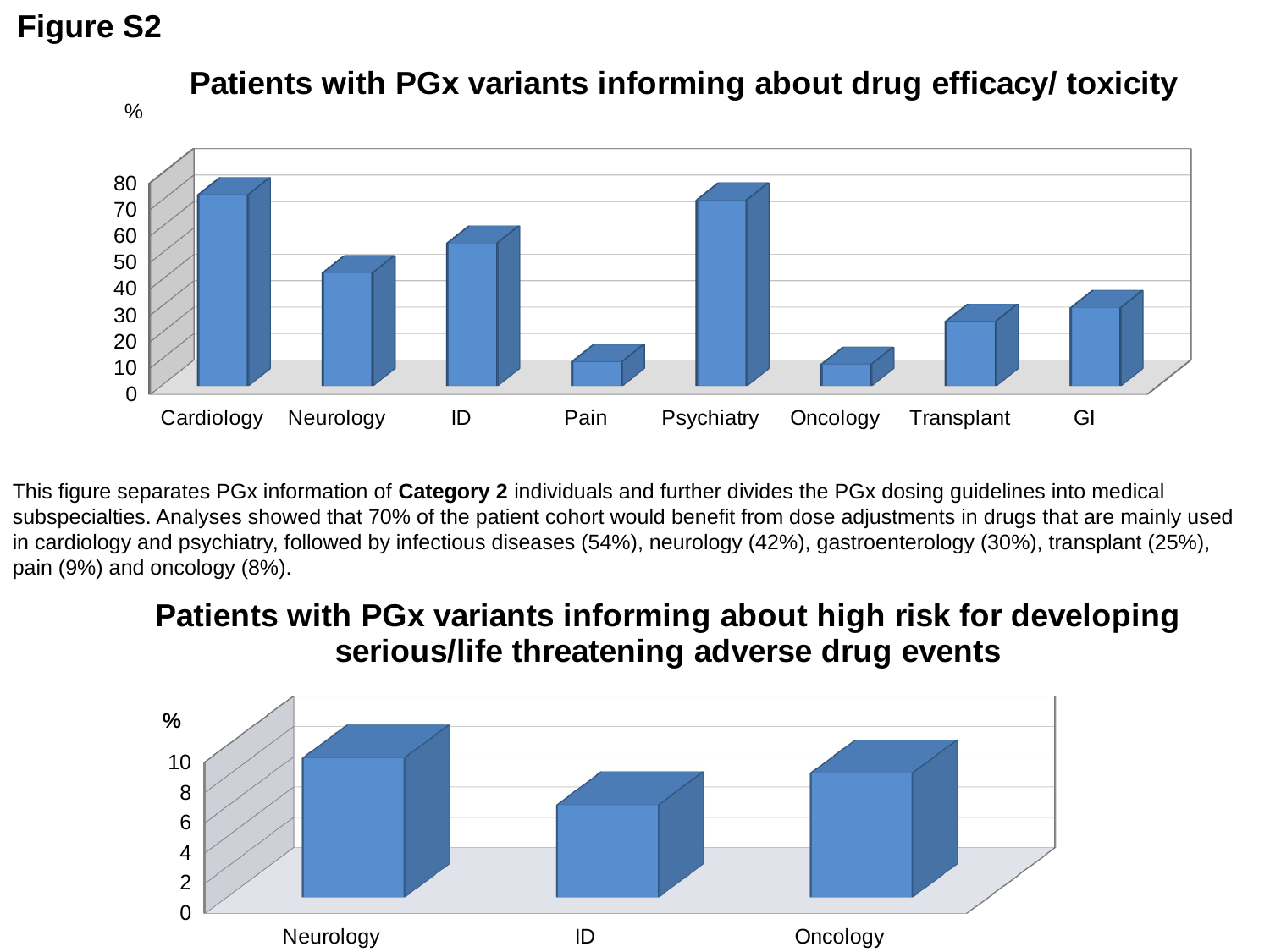

Figure S2
[unsupported chart]
This figure separates PGx information of Category 2 individuals and further divides the PGx dosing guidelines into medical subspecialties. Analyses showed that 70% of the patient cohort would benefit from dose adjustments in drugs that are mainly used in cardiology and psychiatry, followed by infectious diseases (54%), neurology (42%), gastroenterology (30%), transplant (25%), pain (9%) and oncology (8%).
[unsupported chart]
