# Supplementary material for: Genome sequencing as a platform for pharmacogenetic genotyping: a pediatric cohort study
Source: NPJ Genom Med. 2017 May 26;2:19. doi: 10.1038/s41525-017-0021-8 (PMC5677914; doi:10.1038/s41525-017-0021-8)
Supplement: Supplementary file 3 — Supplementary Table 1 [file 41525_2017_21_MOESM3_ESM.doc]

| **Patient ID**  **Table S1: *CYP2D6* CNV, haplotype and phenotype prediction with WGS, WES and MassArray platforms** | **CNV iPLEX** | **CNV WGS** | **Mass Array (iPLEX)** | **WGS** | **WES** | **Phenotype Prediction MassArray (iPLEX)** | **Phenotype Prediction WGS** | **Phenotype Prediction WES** |
| --- | --- | --- | --- | --- | --- | --- | --- | --- |
| **1014** | 2 | 2 | *17/*17 | *17/*17 |  | EM | ^EM |  |
| **1015** | 2 | 2 | *1/*3 | *1/*3 |  | EM | ^EM |  |
| **1016** | 2 | 2 | *41/*41 | *41/*41 | *41/*41 | EM | EM | EM |
| **1001** | 2 | 2 | *4/*9 | *4/*9 |  | IM | ^IM |  |
| **1003** | 2 | 2 | *1/*2 | *1/*2 |  | EM | EM |  |
| **1004** | 2 | 2 | *1/*1 | *1/*1 |  | EM | ^EM |  |
| **1005** | 2 | 2 | *1/*2 | *1/*2 |  | EM | ^EM |  |
| **1006** | 2 | 2 | *1/*35 | *1/*35 |  | EM | ^EM |  |
| **1007** | 2 | 2 | *2/*2 | *2/*2 |  | EM | EM |  |
| **1008** | 2 | 2 | *2/*10 | *2/*10 | *2/*10 | EM | ^EM | EM |
| **1009** | 2 | Inconclusive | *1/*4 | *1/*4 | *1/*4 | EM | ^EM | EM |
| **1013** | 2 | 2 | *1/*2 | *1/*2 |  | EM | EM |  |
| **1000** | 2 | 2 | *1/*4 | *1/*4 |  | EM | ^EM |  |
| **1002** | 2 | 2 | *1/*2 | *1/*2 |  | EM | ^EM |  |
| **1011** | 2 | 2 | *1/*6 | Most similar to *1/*6 | *1/*6 | EM | *^EM* | EM |
| **1012** | **3** | **3** | *2/*10 | *2/*10 | *2/*10 | *EM/UM?* | ^UM^1^ | EM |
| **1018** | **3** | **3** | *2/*10 | *2/*10 |  | *EM/UM?* | ^UM^1^ |  |
| **1019** | 2 | 2 | *1/*4 | *1/*4 |  | EM | EM |  |
| **1021** | 2 | 2 | *2/*4 | *2/*4 |  | EM | ^EM |  |
| **1024** | 2 | 2 | *1/*41 | *1/*41 |  | EM | ^EM |  |
| **1029** | **3** | **3** | *2/*41 | *2/*41 | *2/*41 | *EM/UM?* | EM^1^ | EM |
| **1034** | 2 | 2 | *2/*41 | *2/*41 |  | EM | EM |  |
| **1035** | 2 | 2 | *2/*2 | *2/*2 |  | EM | EM |  |
| **1036** | 2 | 2 | *2/*41 | *2/*41 |  | EM | EM |  |
| **1038** | 2 | 2 | *4/*41 | *4/*41 |  | IM | ^IM |  |
| **1052** | 2 | 2 | *1/*4 | *1/*4 |  | EM | EM |  |
| **1010** | 2 | 2 | *1/*41 | *1/*41 |  | EM | ^EM |  |
| **1020** | **3** | **3** | *1/*1 | *1/*1 |  | UM | UM |  |
| **1023** | 2 | 2 | *1/*3 | *1/*3 | *1/*3 | EM | EM | EM |
| **1026** | 2 | 2 | *1/*1 | *1/*1 |  | EM | ^EM |  |
| **1027** | 2 | 2 | *1/*4 | Most similar to *1/*4 |  | EM | *^EM* |  |
| **1028** | 2 | 2 | *1/*1 | Most similar to *1/*1 |  | EM | *Unknown* |  |
| **1030** | 2 | 2 | *1/*2 | *1/*2 |  | EM | EM |  |
| **1031** | 2 | Inconclusive | *1/*4 | Most similar to *1/*4 |  | EM | *^Unknown* |  |
| **1032** | 2 | 2 | *1/*35 | *1/*35 |  | EM | ^EM |  |
| **1039** | **1** | **1** | *2/*5 | Most similar to *2/*5 |  | EM | *^Unknown* |  |
| **1040** | 2 | 2 | *1/*2 | *1/*2 |  | EM | ^EM |  |
| **1042** | 2 | 2 | *2/*2 | *2/*2 |  | EM | EM |  |
| **1043** | 2 | Inconclusive | *4/*7 | *4/*7 |  | PM | ^PM |  |
| **1044** | 2 | 2 | *2/*6 | *2/*6 |  | EM | EM |  |
| **1045** | 2 | 2 | *1/*2 | *1/*2 |  | EM | EM |  |
| **1046** | 2 | 2 | *1/*7 | *1/*7 |  | EM | EM |  |
| **1048** | 2 | 2 | *1/*17 | Most similar to *1/*17 |  | EM | *^EM* |  |
| **1049** | 2 | 2 | *2/*41 | *2/*41 |  | EM | EM |  |
| **1050** | 2 | 2 | *1/*1 | Most similar to *1/*1 |  | EM | *^EM* |  |
| **1051** | 2 | 2 | *2 /*2 | **2/*2* |  | EM | EM |  |
| **1053** | **1** | **1** | *1/*5 | *Unknown* |  | EM | *^Unknown* |  |
| **1055** | 2 | 2 | *2/*2 | *2/*2 | *2/*2 | EM | EM |  |
| **1022** | 2 | 2 | *1/*4 | Most similar to *1;*7/*4 |  | EM | *^Unknown* |  |
| **1047** | 2 | 2 | *1/*2 | *1/*2 |  | EM | EM |  |
| **1057** | 2 | 2 | *1/*4 | *1/*4 |  | EM | ^EM |  |
| **1058** | **3** | **3** | *4/*4 | *4/*4 |  | PM | ^PM |  |
| **1059** | 2 | 2 | *1/*41 | *1/*41 |  | EM | EM |  |
| **1060** | 2 | 2 | *1/*2 | *1/*2 |  | EM | ^EM |  |
| **1061** | **4** | **4** | *2/*2 | *2/*2 |  | UM | UM |  |
| **1063** | **1** | **1** | *2/*5 | Most similar to *2/*2 |  | EM | *Unknown* |  |
| **1066** | 2 | 2 | *4/*4 | Most similar to *4/*4 |  | PM | *^Unknown* |  |
| **1067** | 2 | 2 | *1/*35 | Most similar to *1/*2 |  | EM | *EM* |  |
| **1068** | 2 | Inconclusive | *4/*41 | *4/*41 |  | IM | *^Unknown* |  |
| **1070** | 2 | Inconclusive | *1/*4 | Most similar to *1/*4 |  | EM | *^EM* |  |
| **1071** | 2 | 2 | *2/*2 | *2/*2 |  | EM | EM |  |
| **1072** | 2 | Inconclusive | *1/*4 | Most similar to *1/*4 |  | EM | *^EM* |  |
| **1073** | 2 | 2 | *1/*4 | Most similar to *1/*4 |  | EM | *^EM* |  |
| **1076** | 2 | 2 | *1/*1 | *1/*1 |  | EM | ^EM |  |
| **1078** | 2 | 2 | *1/*2 | *1/*2 |  | EM | EM |  |
| **1081** | 2 | 2 | *2/*41 | *2/*41 |  | EM | EM |  |
| **1082** | 2 | 2 | *2/*35 | *2/*35 |  | EM | EM |  |
| **1062** | 2 | 2 | *4/*35 | *4/*35 |  | EM | ^EM |  |
| **1064** | 2 | 2 | *1/*1 | *1/*1 |  | EM | ^EM |  |
| **1079** | 2 | 2 | *2/*41 | *2/*41 |  | EM | EM |  |
| **1080** | 2 | 2 | *2/*41 | *2/*41 |  | EM | EM |  |
| **1065** | **1** | **1** | *2/*5 | *2/*2 | *2/*2 | EM | EM |  |
| **1074** | **1** | Inconclusive | *2/*5 | Most similar to *2/*2 |  | EM | *^EM* |  |
| **1075** | **3** | 2 | *1/*4 | Most similar to *1/*4 |  | EM | *Unknown* |  |
| **1083** | 2 | 2 | *1/*35 | Most similar to *1/*2;*35 |  | EM | *^EM* |  |
| **1084** | 2 | 2 | *1/*2 | *1/*2 |  | EM | EM |  |
| **1085** | **3** | **3** | *1/*1 | *1/*1 |  | UM | ^UM |  |
| **1086** | **1** | **1** | *1/*5 | Most similar to *1/*1 |  | EM | *^Unknown* |  |
| **1088** | 2 | **1** | *29/*29 | *5/*29 |  | EM | IM |  |
| **1089** | 2 | 2 | *1/*41 | Most similar to *1/*41 | *1/*41 | EM | *^EM* |  |
| **1090** | **1** | **1** | *1/*5 | Most similar to *1/*1 |  | EM | *^Unknown* |  |
| **1091** | 2 | 2 | *1/*41 | Most similar to *1/*41 |  | EM | *^EM* |  |
| **1092** | 2 | 2 | *4 /*10;*36 | *Unknown* |  | IM | *^Unknown* |  |
| **1093** | **3** | **3** | *1/*4 | Most similar to *1/*4 |  | EM | *^Unknown* |  |
| **1041** | 2 | 2 | *2/*41 | *2/*41 |  | EM | EM |  |
| **1096** | **3** | **3** | *1/*10 or *39/*68 | *1/ *Unknown* |  | *EM/UM?* | *^Unknown* |  |
| **1097** | 2 | 2 | *1/*9 | *1/*9 | *1/*9 | EM | ^EM |  |
| **1099** | 2 | 2 | *1/*1 | *1/*1 |  | EM | EM |  |
| **1100** | 2 | 2 | *1/*2 | *1/*2 |  | EM | ^EM |  |
| **1102** | 2 | 2 | *1/*2 | Most similar to *1/*2 |  | EM | *^EM* |  |
| **1105** | 2 | 2 | *1/*1 | *1/*1 |  | EM | EM |  |
| **1106** | 2 | 2 | *1/*1 | *1/*1 |  | EM | ^EM |  |
| **1108** | 2 | 2 | *1 /*4B | Most similar to *1/*4;*12 |  | EM | *^Unknown* |  |
| **1112** | 2 | Inconclusive | *4/*10 | Most similar to *4/*10 |  | IM | *^Unknown* |  |
| **1094** | 2 | 2 | *1/*4 | Most similar to *1/*4 |  | EM | *^EM* |  |
| **1025** | 2 | Inconclusive | *4 / *4 | *4/*4 |  | PM | ^PM |  |
| **1056** | 2 | 2 | *2/*10 | *2/*10 |  | EM | ^EM |  |
| **1103** | *2* | *2* | **1/*2* | *most similar to *1/*2* |  | *EM* | *^EM* |  |

| ***EM****= extensive (normal) metabolizer* |
| --- |
| ***IM****= intermediate metabolizer* |
| ***PM****= poor metabolizer* |
| ***UM****= ultra-rapid metabolizer* |
| ***^****=**missing* ***rs16947*** |

| **^1^Patient ID** | Copy Number Variant | Read count | Haplotype |
| --- | --- | --- | --- |
| **1012** | 3 | rs1080985: 79:50,29 | *2/*2/*10 |
| **1018** | 3 | rs1080985: 75: 50,25 | *2/*2/*10 |
| **1029** | 3 | rs1080985: 87:25,62  rs28371725: 64:24,40 | *2/*41/*41 |
