# Supplementary material for: Genome sequencing as a platform for pharmacogenetic genotyping: a pediatric cohort study
Source: NPJ Genom Med. 2017 May 26;2:19. doi: 10.1038/s41525-017-0021-8 (PMC5677914; doi:10.1038/s41525-017-0021-8)
Supplement: Supplementary file 4 — Supplementary Table 2 [file 41525_2017_21_MOESM4_ESM.doc]

| **Reference SNP** | **Gene** | **Discordant Reads** | **Number of samples** |
| --- | --- | --- | --- |
| **rs1799853** | *CYP2C9* | TT vs CT | 1 |
| **rs12979860** | *IFNL3* | TT vs CT | 4 |
| **rs1061235** | *HLA-A*3101* | AA vs AT | 1 |

**Table S2:** Differences in genomic variant identification between WGS and MassArray platform in 6 subjects
