# Supplementary material for: Genome sequencing as a platform for pharmacogenetic genotyping: a pediatric cohort study
Source: NPJ Genom Med. 2017 May 26;2:19. doi: 10.1038/s41525-017-0021-8 (PMC5677914; doi:10.1038/s41525-017-0021-8)
Supplement: Supplementary file 5 — Supplementary Table 3 [file 41525_2017_21_MOESM5_ESM.doc]

**Table S3: *CYP2D6* additional variants extracted from WGS data**

| **hg19Co-**  **ordinates**  **in Chr22**  **Co-** | **Ref-**  **er-**  **ence** | **Geno-**  **type** | **Read**  **Count**  **Allele1** | **Read**  **Count**  **Allele2** | **ExAc**  **Frequency** | **dbsnp** | **Sample** | **Effect**  **NM_**  **000106** | **Asso-**  **ciated**  ***CYP2D6***  **Allele** | ***CYP2D6* e by targeted**  **panel**  **result** | **Missense**  **Variant**  **Pre-**  **dictions** | **Meta-**  **bolizer**  **status**  **change** |
| --- | --- | --- | --- | --- | --- | --- | --- | --- | --- | --- | --- | --- |
| **42526775** | C | C|T | 20 | 19 | 0.001873 | rs72549358 | 1102 | V7M | *28 | *1/*2 | tolerated | No |
| **42525089** | G | G|C | 20 | 15 | 0.002182 | rs78482768 | 1102 | Q151E | *28 | *1/*2 | tolerated | No |
| **42526717** | C | C|T | 16 | 11 | 0.002496 | rs28371696 | 1076 1026 1048 | R26H | *43 | *1/*1 or *1/*17 | tolerated | No |
| **42526712** | G | G|A | 18 | 21 | 0.002487 | rs138100349 | 1105 | R28C | *22 | *1/*1 | dele-  terious | No |
| **42526672** | G | G|A | 7 | 10 | 0.000008148 | rs373243894 | 1038 | P41L | n/a | *4/*41 | dele-terious | Possibly |
| **42525781** | A | A|T | 10 | 17 | 0.00004909 | rs76187628 | 1044 | V104E | n/a  but V104A and V104M observed | *2/*6 | tolerated | No |
| **42524323** | A | A|G | 15 | 10 | 0.007515 | rs17002852 | 1081 | H232H |  | *2/*41 | n/a | No |
| **42524218** | G | G|T | 26 | 19 | 0.01 | rs28371718 | 1108 | P267P |  | *1/*4 | n/a | No |
| **42524191** | C | C|A | 21 | 20 | 0.003788 | rs28371719 | 1048 | L276L |  | *1/*17 | cryptic acceptor activated? | Unknown |
| **42523854** | GGA | GGA|AGG | 25 | 50 | 0.0002484, 0.0002981 | rs28371724, rs140513104 | 1043 | P325L | *4G | *4/*7 | dele-terious | No |
| **42523844** | G | G|A | 7 | 9 | 0.000171 | rs150216909 | 1035 | R329C |  | *2/*2 | dele-terious | No |
| **42523636** | C | C|A | 23 | 45 | 0.074 | rs3915951 | 1009 | R329L |  | *1/*4 | dele-terious | Possibly |
| **42522601** | T | T|C | 30 | 39 | 0.0003583 | rs199722016 | 1076 | Y490C |  | *1/*1 | dele-terious | No |
